# Evaluating cannabidiol (CBD) expectancy effects on acute stress and anxiety in healthy adults: a randomized crossover study

**DOI:** 10.1007/s00213-021-05823-w

**Published:** 2021-04-04

**Authors:** Toni C. Spinella, Sherry H. Stewart, Julia Naugler, Igor Yakovenko, Sean P. Barrett

**Affiliations:** 1 Department of Psychology and Neuroscience, Dalhousie University, Life Sciences Centre, 1355 Oxford Street, PO Box 15000, Halifax, Nova Scotia B3H 4R2 Canada; 2 Department of Psychiatry, Dalhousie University, Life Sciences Centre, 1355 Oxford Street, PO Box 15000, Halifax, Nova Scotia B3H 4R2 Canada

**Keywords:** Cannabidiol, CBD, Cannabis, Expectancy, Placebo, Stress, Anxiety, Affect, Anxiolytic, Subjective response

## Abstract

**Rationale:**

Cannabidiol (CBD) has been reported to attenuate stress and anxiety, but little is known about the extent to which such effects result from pharmacological versus expectancy factors.

**Objectives:**

We evaluated whether CBD expectancy alone could influence stress, anxiety, and mood, and the extent to which beliefs regarding CBD effects predicted these responses.

**Methods:**

In this randomized crossover study, 43 health adults (23 women) attended two experimental laboratory sessions, where they self-administered CBD-free hempseed oil sublingually. During one session, they were (incorrectly) informed that the oil contained CBD and in the other session, that the oil was CBD-free. Following administration, participants engaged in the Maastricht Acute Stress Test (MAST). Heart rate variability (HRV) was assessed continuously, and subjective state was assessed at baseline, 90-min following oil administration, immediately following the MAST, and after a 10-min recovery period.

**Results:**

The CBD expectancy condition was associated with increased sedation as well as with changes in HRV that were consistent with heightened anticipatory stress regulation. Overall, there were no systematic changes in subjective stress, or anxiety, according to expectancy condition. However, participants who endorsed strong *a priori* beliefs that CBD has anxiolytic properties reported significantly diminished anxiety in the CBD expectancy condition.

**Conclusions:**

CBD expectancy alone impacted several subjective and physiological responses. Additionally, expectancy-related factors were implicated in anxiolytic effects of CBD for those who believed it was helpful for such purposes, emphasizing the need to measure and control for CBD-related expectancies in clinical research that involves the administration of CBD.

**Supplementary Information:**

The online version contains supplementary material available at 10.1007/s00213-021-05823-w.

## Introduction

In the past decade, there have been notable increases in cannabidiol (CBD) use globally for therapeutic purposes (World Health Organization (World Health Organization (WHO) 2018). One potential application that has generated considerable interest is for the treatment of stress- and anxiety-related disorders. In animal models, CBD has been shown to diminish several anxiety- and stress-related responses (Blessing et al. 2015), while in humans, CBD’s effect on stress and anxiety has been somewhat mixed. For example, while one study found that CBD had less of an impact on anxiety symptoms relative to placebo in a sample of adults with obsessive-compulsive disorder (Kayser et al. 2020), other investigations have found that CBD reduces the anxiogenic effects induced by THC (Zuardi et al. 1982), and attenuates anxiety associated with social stress in both healthy individuals (Zuardi et al. 1993, 2017) and individuals with social anxiety disorder (Bergamaschi et al. 2011; Masataka 2019). However, the extent to which any such anxiolytic effects result from the pharmacological properties of CBD, and/or CBD-related expectancy, has never been systematically examined.

Drug effects in humans are believed to be comprised of both direct pharmacological effects related to the drug itself and a placebo response (Kirsch 1985). The placebo effect is thought to be mediated by the patient’s beliefs or expectations regarding the content and effects of a substance. Indeed, such expectations can be formed by verbal information about the content and supposed effects of a substance, prior experience, and observational learning (Kirsch 2018). Evidence suggests that placebo responses may account for a significant portion of the therapeutic response to drugs such as nicotine replacement therapies (Dar and Barrett 2014), antidepressants (Laferton et al. 2018), and analgesics (Klinger et al. 2018). Though active treatment versus placebo study designs can control for some of the influence of non-pharmacological variables, it is not possible to completely disentangle placebo effects from pharmacologically driven treatment effects using such designs (Wampold et al. 2005; Lund et al. 2014). Thus, given the current state of research to our knowledge, CBD-related placebo responses have never been systematically examined. If a placebo effect is observed for CBD, it would bolster the case for future evaluation of whether CBD pharmacology interacts with expectancy to dampen stress- and anxiety-related responses using a full balanced placebo design (Rohsenow and Marlatt 1981).

We designed the present randomized crossover study to evaluate whether CBD expectancy, independent from pharmacology, could impact acute stress, anxiety, and mood responses to a standardized stressor in a sample of healthy adults. Following one orientation session, subjective and physiological data were gathered at numerous time points throughout two experimental laboratory sessions in the context of a validated stress induction protocol. In terms of physiological measures, heart rate (HR), and heart rate variability (HRV) were chosen as indices of stress and anxiety. The root mean square successive difference (RMSSD) is a widely used index of HRV that is thought to reflect parasympathetic output and successful emotional regulation (Laborde et al. 2017). Thus, lower mean RMSSD is thought to indicate a larger stress response. Additionally, we sought to examine the extent to which individual differences in beliefs regarding CBD effects (i.e., response expectancies) are activated to influence responses to perceived CBD vs. perceived placebo administration. Consistent with prior expectancy research (e.g., Klinger et al. 2018; Laferton et al. 2018), we hypothesized that the CBD expectancy condition would be associated with distinct patterns of subjective and physiological responses relative to the CBD-free expectancy condition. Specifically, we expected the CBD expectancy condition would be associated with lower levels of subjective and physiological indices of stress, anxiety, and negative affect. Additionally, response expectancies tend to be self-confirming such that simply expecting a specific response to occur (e.g., anxiety reduction) will enhance the likelihood of said response to actually occur (Kirsch 1985). As such, it was also hypothesized that endorsing stronger beliefs regarding the potential stress-, anxiety-, and mood-related benefits of CBD would be linked to the largest differences in the corresponding subjective responses between the two conditions.

## Methods

### Study design and participants

We conducted a three-session (one orientation session, two experimental sessions), within-subjects experimental laboratory study with healthy adults who were community-recruited from the Halifax Regional Municipality (Nova Scotia, Canada). We were unable to conduct an *a priori* power analysis for our particular analytic approach (generalized estimating equations) due to our within-subject study design parameters. To address this barrier, we conducted a power calculation after data collection using a similar, but less powerful analytic approach (repeated-measure ANOVA) in G*Power. Based on aggregated effect sizes of placebo effects for conditions that are very amenable to the placebo effect (e.g., anxiety; *d* = .29; Wampold et al. 2005), we are expected to have at least 85% power to detect within-factor effects with the sample size used in this study. As such, we expected to have sufficient statistical power to test our study hypotheses (i.e., main effects, two-way interactions).

Participants were required to be at least 19 years of age, as this is the age of majority in Nova Scotia. Selection criteria included ≥ 1 lifetime uses of cannabis, which was required to ensure that subjects had some experience with and knowledge about cannabis in attempt to standardize expectations to some extent. To help ensure that participants could meet the abstinence requirements, only individuals reporting cannabis use two or fewer days per week in the past month were enrolled in the study. In order to ensure cold pressor test (CPT) would be well-tolerated, participants were required to be medically healthy, and free of any serious medical conditions, or any history of fainting, seizures, circulatory disorders, heart problems, high blood pressure, diabetes, frostbite, or any current cut, sore, or fracture to their right hand/arm (Mitchell et al. 2004; Birnie et al. 2011). Subjects were also excluded if they reported current prescription medication use (except birth control in females) or any current psychiatric disorder, as diagnosed by a health care professional, including substance use disorders (American Psychiatric Association 2013). These exclusions helped prevent pre-existing neurophysiological or psychological conditions from influencing subjective and physiological stress, mood, and anxiety responses to the laboratory stressor. Participants were also required to be cannabis oil naïve (to enhance believability of the oil manipulation), and to have never previously participated in a study conducted by our group that involved deception.

### Stress and anxiety induction

The Maastricht Acute Stress Test (MAST; Smeets et al. 2012) was used to induce stress and state-anxiety in our sample. The MAST was chosen since it possesses both physical and psychological features that have been demonstrated to reliably provoke subjective and physiological responses associated with stress and anxiety in laboratory settings (Smeets et al. 2012; Bali and Jaggi 2015) over multiple sessions, with little habituation (Quaedflieg et al. 2017). The physical feature is a CPT and the psychological feature mental arithmetic challenges that include a psychosocial evaluative threat (Smeets et al. 2012; Bali and Jaggi 2015).

As per the validated protocol (Smeets et al. 2012), the MAST involved a 5-min anticipation phase, in which instructions and procedures are explained to participants, followed by a 10-min acute stress phase. During the stress phase, participants engaged in trials alternating between (i) immersing their hand into ice-cold water (2 °C) (i.e., CPT), and (ii) counting backwards in steps of 17 or 13 starting at a random four-digit number. Both tasks are combined with negative social-evaluative pressure (i.e., negative feedback and videotaping).

### Measures

#### Physiological measures

Electrocardiogram (ECG) data was collected continuously throughout the experimental sessions using an Equivital EQ02 sensor electronic module (SEM) equipped to a fitted Life Monitor belt (ADInstruments; [ADI], Colorado Springs, USA). The EQ02 device measured ECG signal on two channels via three electrodes at a sampling rate of 1000 Hz. The raw ECG signals were later transferred to a computer and used to compute indices of heart rate and HRV, a robust, non-invasive physiological measure that has been used to assess stress and anxiety responses (Kim et al. 2018).

#### Demographics and CBD belief ratings

Demographic information, including age, sex, ethnicity, and level of education, were collected with a researcher-compiled self-report questionnaire. Additionally, information about participants’ baseline/*a priori* beliefs regarding the effects of CBD were collected using three researcher-compiled single-item questions. Participants reported on the extent to which they believed statements about the mood-, stress-, and anxiety-related properties of CBD (i.e., “improves mood,” “reduces stress “reduces anxiety”) on a 10-point scale (1- “Not at all,” 10- “Completely”). Lifetime and past month cannabis use frequency information was collected using single items via telephone screening as part of the study selection criteria.

#### Subjective stress, anxiety, mood, and drug effect ratings

Participants reported their current subjective state using a combination of validated measures and researcher-compiled single-item scales. Subjective stress was assessed with a single-item Numerical Rating Scale (NRS) where subjects rated the extent to which they felt “stressed” on a 10-point scale (1- “Not at all,” 10- “Extremely”). Similar single-item scales have been shown to demonstrate adequate construct validity (correlations between .45 and .66 with other validated stress measures) and discriminant validity (i.e., stressed vs. non-stressed states) (Lesage et al. 2012).

Subjective anxiety was assessed with a six-item shortened state version of the State-Trait Anxiety Inventory (STAI-S-SF; Marteau and Bekker 1992). Participants rated six statements about their current state (e.g., “I am tense”) and rate them on a 4-point scale (1- “Not at all,” 4- “Very much”). The STAI-S-SF has been shown to possess good reliability (*α* = .82; Marteau and Bekker 1992). It also produces acceptable validity, generating similar scores to those obtained using the full 20-item STAI-S (Spielberger 1983) (.91 total score correlation; Marteau and Bekker 1992), which is sensitive to rapid state-dependent fluctuations in anxiety (Rossi and Pourtois 2012). To calculate total anxiety scores, the three positive STAI-S-SF items were first reverse scored. Next, all scores were summed then multiplied by 20/6 to yield total scores between 20 and 80.

Subjective mood was assessed with the ten-item International Positive and Negative Affect Schedule, Short Form (I-PANAS-SF; Thompson 2007). Participants were asked to rate the extent to which they presently feel a list of positive affect (e.g., “Alert,” “Inspired”) and negative affect (e.g., “Upset”, “Hostile”)-related items on a 5-point scale (1- “Very slightly or not at all,” 5- “Extremely”). Both positive and negative affect subscales of the I-PANAS-SF have been shown to possess adequate reliability (*α* = .78 and .76, respectively), as well as acceptable convergent validity with measures of subjective well-being (Thompson 2007). To calculate total scores, the five items from each subscale within the I-PANAS-SF were summed to create a positive affect and negative affect score (subscale scores range between 5 and 25).

Subjective drug effects were assessed using the six-item Brief Biphasic Alcohol Effects Scale (B-BAES; Rueger et al. 2009). Participants rated how well three sedation items (e.g., “Sedated”) and three stimulation items (e.g., “Energized”) described their current feelings on a 10-point scale (1- “Not at all,” 10- “Extremely”). The subscales within the B-BAES correlated highly (.92–.97) with the full form of the BAES (Martin et al. 1993), demonstrating adequate criterion validity, and showed excellent internal consistency reliability (*α* = .89–.91; Rueger and King 2013). Though the B-BAES was initially developed to evaluate the biphasic stimulation and sedation effects associated with alcohol use, the questions are not specific to alcohol and thus were used to assess subjective sedation- and stimulation-related drug effects in this study. To calculate total scores, the three items from each of the two subscales within the B-BAES were summed to create a sedation and stimulation score (subscale score range between 10 and 30). Additionally, two researcher-compiled NRS items (“intoxicated,” “relaxed”) rated on a 10-point scale (1- “Not at all,” 10- “Extremely”) were included in the assessment of potential drug-related effects.

### Procedures

Once eligibility was confirmed via telephone screening, participants were scheduled for an initial orientation session (~ 30 min). After providing consent, participants had their weight measured, which they were informed would determine the dose of oil that they would be given during their experimental sessions. Demographic and CBD belief rating information was collected. Two experimental sessions were then scheduled between 10:30 and 18:00 h. The laboratory setting, testing procedure, and time of day was kept constant for each participant across all sessions to minimize circadian fluctuations in the stress response (Nicolson and van Diest 2000). Female participants not using birth control were tested during the luteal phase of their menstrual cycle to minimize menstrual cycle-related fluctuations in the stress response (Barel et al. 2018). Experimental sessions were separated by a minimum of 1 week and a maximum of 1 month.

All participants received CBD-free hemp seed oil across both experimental sessions but received different instructions during each session about the CBD content of the oil (told CBD-containing vs. told CBD-free), in randomized order. This produced two conditions: (a) told CBD, administered CBD-free; (b) told CBD-free, administered CBD-free, allowing for an assessment of the effects of CBD-related expectancy, independent from pharmacology (Sutton 1991). Experimenters were blind to the expectancy condition, as oil was administered by an independent blinder who otherwise did not interact with the participant, and participants were blind to the actual pharmacology of the oil.

Experimental sessions (~ 3 h) took place following a minimum of 72 h of abstinence from cannabis, given the ~ 31 h half-life of CBD and THC in infrequent users (Smith-Kielland et al. 1999; Millar et al. 2018). Additionally, 12 h of abstinence from alcohol, tobacco smoking, and other drug consumption was required (Holford 1987; Benowitz and Jacob 1994). Participants were also required to abstain from caffeine for a minimum of 2 h (Benowitz et al. 1995) as well as to fast for 1 h prior to their session. Abstinence from substances was verified via self-report since all participants were pre-screened to be healthy, infrequent cannabis users with no current substance dependencies. To increase compliance to the study procedures, participants were sent multiple email reminders about their upcoming experimental sessions and the respective abstinence requirements.

Following the collection of baseline subjective and HRV data, participants were administered hemp seed oil sublingually by an independent blinder. To enhance the believability of the drug content instructions, participants were presented with their assigned oil in packaging consistent with the instructions provided. Participants were informed during the consent process, and by the independent blinder, there would be a 90-min “absorption period” following oil administration (to mimic the absorption period of CBD; Zuardi et al. 2017). During this period, participants were provided with neutral word puzzles and reading material to pass the time. For the second time (post-absorption), participants completed the same battery of assessments as used at baseline. To induce stress and state-anxiety, the MAST protocol was administered by the experimenter. Immediately following completion of the MAST, subjective measures were re-administered for the third time (post-stress), and for a final time 10 min later (recovery).

At the end of each experimental session, participants were asked about the CBD content of the oil that they had self-administered, with the following response options “CBD oil,” “CBD-free/hemp seed oil,” or “Unsure.” This served as a manipulation check to determine whether participant beliefs regarding drug assignment were consistent with the CBD content stated in their instructions. It was decided *a priori* that sessions where participant did not believe the CBD content information provided would be excluded from the analyses to avoid confounding the interpretation of results. Lastly, to ensure that the deceptive nature of the study was not revealed to potential future participants, full debriefing of the nature and aims of the study was delayed until study data collection concluded.

### Data acquisition and ECG pre-processing

Raw ECG data were extracted from Lead II with LabChart Pro software (HRV 2.0 module; ADI). HRV was calculated by extracting beat-to-beat RR intervals. Ectopic beats were excluded from analyses, using the Lomb-Scargle Periodogram, to enable exclusion of ectopic beats without interpolation. To reduce baseline wandering, all ECG signals were passed through a high pass filter (.5 Hz). All segments used in analyses were visually inspected for ectopic beats and noise. If noise or ectopic beats exceeded 5% of total beats in an ECG segment, they were excluded. An artifact-free 5-min segment during the first 70 min of the session was selected as a baseline. A 5-min segment was selected during the anticipation phase of the MAST (anticipation), and the two 5-min segments during the arithmetic and cold pressor components of the MAST were averaged to compute HRV during acute stress (stress). The final 5-min segment was selected 10 min after the MAST (recovery). Additionally, an ECG-derived respiration rate (EDR) was manually calculated from raw Lead II ECG as per recommendations (Brugnera et al. 2018).

Heart rate (HR) and the root mean square successive difference (RMSSD), a time-domain index of HRV, were extracted from the RR data. RMSSD is a widely used index of HRV that is thought to reflect parasympathetic output and successful emotional regulation (Laborde et al. 2017).

### Statistical analyses

Statistical analyses were conducted in SPSS, version 25.0. Generalized estimating equations (GEE) were used for all primary analyses because they have robust estimators and can accommodate missing data as well as non-normal distributions (Hubbard et al. 2010). Multiple models per outcome were conducted to determine the optimal fit for the data based on the lowest number of parameters and the lowest Quasi Likelihood under Independence Model Criterion (QIC). First, the dependent variable was visually screened to identify plausible distributions, which were then compared with the covariate structure specified as Unstructured. Once an optimal distribution was chosen, plausible covariate structures were tested. The Exchangeable correlation matrix tended to be most parsimonious among all models.

Subjective outcomes included stress, state anxiety (STAI-S-SF), mood (I-PANAS-SF positive affect and negative affect), and drug effects (B-BAES sedation and stimulation; intoxication; relaxation). For subjective outcomes, time (baseline, post-absorption, post-stress, recovery), and expectancy condition (CBD, CBD-free) were entered as repeated factors. Physiological outcomes included HR and HRV (i.e., RMSSD). The physiological outcomes were analyzed in a similar fashion to the subjective outcomes. Specifically, Time (baseline, anticipation, stress, recovery) and Expectancy condition (CBD, CBD-free) were entered as repeated factors, and EDR was entered as a covariate to control for respiratory influences on HR and HRV (Brugnera et al. 2018). Effects of interest for subjective and physiological outcomes included main effects of time and interactions between Time and Expectancy condition. Planned post-hoc pairwise comparisons using tests of simple effects were used to probe main effects of time and Time by Expectancy condition interactions. We also examined whether *a priori* beliefs about CBD influenced corresponding stress, anxiety, and mood responses according to expectancy condition. The corresponding CBD belief rating and baseline outcome values were entered as covariates. Time (all time points following oil administration: post-absorption, post-stress, recovery) and expectancy condition (CBD, CBD-free) were entered as repeated factors. Effects of interest included expectancy condition by belief interactions on overall stress, anxiety, and mood ratings. Given that GEE in SPSS does not have the capability of probing interactions involving continuous predictors using tests of simple effects, we used “geepack” in R (version 4.0) to probe significant interactions involving CBD belief rating. Post-hoc tests of simple effects involved contrasts between expectancy condition across three levels of CBD belief ratings (i.e., terciles). All *p*-values less than .05 were considered significant. Additionally, the Benjamini-Hochberg method (Benjamini and Hochberg 1995) was used to control for the false discovery rate (FDR) within each model (i.e., family) tested. The FDR threshold was set at .05 such that there was a 5% chance that any finding within each model was a false discovery. All *p*-values were reported in their original format unless the FDR threshold was exceeded, in which case both adjusted and unadjusted *p*-values were reported.

## Results

Forty-three participants, community-recruited between February 2019 and March 2020, were included in the study (age 19–62 years). Five participants withdrew after one session, but their data was retained from the session they completed. One participant withdrew 6 min into the MAST on their second session; thus, their data was excluded after post-absorption. Among the 302 physiological data points collected, 43 were excluded due to excess ECG noise or artifacts exceeding 5%. Additionally, four sessions contained excess noise during one half (i.e., 5 min) of the stress time-point; thus, the mean from the remaining 5-min segment was used to represent acute stress. For subjective data, one case was excluded due to missing data (only for STAI-S-SF). During the manipulation check, all subjects reported oil contents consistent with instructions in 100% of sessions. A summary of participant characteristics is provided in Table 1. All GEE coefficients for interactions, as well as the corresponding estimated marginal means and standard errors are listed in Tables 2 and 3. Main effects of time are reported and described in the Online Resource (Supplemental Table 1). All GEE model coefficients that are not part of the initial hypotheses are reported in the Online Resource (Supplemental Table 2) for descriptive purposes.

**Table 1:** Participant characteristics

	*N* (percent**)**
Age in years (mean (standard deviation))	27.7 (9.3)
Sex	
Female	23 (53.5%)
Male	20 (46.5%)
Females using contraceptives (% of females)	8 (38.4%)
Ethnicity	
Aboriginal and White	3 (7.0%)
White	33 (76.8%)
Black	1 (2.3%)
Latin American	1 (2.3%)
Arab	1 (2.3%)
Southeast Asian, Chinese, or Korean	3 (7.0%)
Other	1 (2.3%)
Highest level of education	
High school diploma	4 (9.3%)
Some college or university	13 (30.2%)
College or university degree	26 (60.5%)
Current (non-dependent) cigarette smoker	2 (4.6%)
Average number of cannabis-using days per week	
0 days	24 (55.8%)
0.5 days	4 (9.3%)
1 days	7 (16.3%)
1.5 days	2 (4.6%)
2 days	6 (14.0%)

*N*, number of subjects

**Table 2:** Estimated marginal mean (standard error) values and generalized estimating equation (GEE) coefficients for Time by Expectancy condition interactions involving subjective drug effects, stress, anxiety, mood, and heart rate variability

Interaction: Time by Expectancy condition								
	Baseline	Post-absorption	Post-stress	Recovery	Outcome	df	Wald Chi-square	*p*
Intoxication					Intoxication	3	7.13	.068
Expect CBD	1.00 (0.01)	1.27 (0.09)	1.35 (0.15)	1.25 (0.10)				
Expect CBD-free	1.01 (0.03)	1.11 (0.07)	1.18 (0.09)	1.13 (0.08)				
Relaxation					Relaxation	3	3.05	.385
Expect CBD	6.21 (0.31)	6.80 (0.33)	2.19 (0.29)	4.95 (0.35)				
Expect CBD-free	6.31 (0.35)	6.26 (0.34)	2.40 (0.29)	4.39 (0.33)				
Stimulation					Stimulation	3	2.15	.542
Expect CBD	14.93 (0.74)	11.65 (1.00)	11.09 (0.94)	12.14 (0.86)				
Expect CBD-free	13.69 (0.76)	11.62 (0.76)	11.63 (0.93)	11.94 (0.85)				
Sedation					**Sedation**	**3**	**16.57**	**.001**
Expect CBD	7.29 (0.57)	9.93 (0.96)	8.12 (0.71)	7.61 (0.76)				
Expect CBD-free	7.81 (0.68)	7.39 (0.56)	8.24 (0.94)	6.36 (0.54)				
Stress					Stress	3	4.50	.212
Expect CBD	2.33 (0.25)	1.65 (0.14)	4.69 (0.40)	1.82 (0.19)				
Expect CBD-free	2.07 (0.27)	1.79 (0.18)	5.01 (0.43)	1.98 (0.20)				
Anxiety					Anxiety	3	5.04	.169
Expect CBD	31.04 (1.23)	29.64 (1.21)	54.62 (1.79)	36.35 (1.52)				
Expect CBD-free	31.45 (1.48)	33.38 (1.53)	56.04 (1.66)	38.45 (1.54)				
Negative affect					Negative affect	3	.23	.973
Expect CBD	5.75 (0.16)	5.63 (0.19)	9.32 (0.54)	6.17 (0.25)				
Expect CBD-free	6.06 (0.22)	6.01 (0.23)	9.63 (0.56)	6.37 (0.25)				
Positive affect					Positive affect	3	1.88	.599
Expect CBD	13.32 (0.62)	10.99 (0.72)	12.24 (0.67)	11.82 (0.63)				
Expect CBD-free	12.91 (0.57)	11.60 (0.54)	12.64 (0.70)	12.11 (0.59)				
	Baseline	Anticipation	Stress	Recovery				
HR					HR	3	2.55	.466
Expect CBD	68.86 (1.20)	71.69 (1.13)	75.94 (1.68)	63.86 (1.18)				
Expect CBD-free	68.45 (1.26)	72.47 (1.80)	74.07 (1.60)	63.21 (1.31)				
RMSSD					**RMSSD**	**3**	**8.09**	**.044**
Expect CBD	59.59 (6.03)	70.41 (4.85)	54.59 (3.80)	81.05 (5.64)				
Expect CBD-free	62.68 (5.97)	62.60 (5.00)	61.88 (4.90)	81.61 (5.93)				

Bolded coefficients indicate statistical significance (*p* < .05)

Subjective measures: Baseline (T1): +00; Post-absorption (T2): +95; Post-stress (T3): +110; Recovery (T4): +120

Physiological measures: Baseline (T1): +00 − + 70; Anticipation (T2): +95; Stress (T3): +100; Recovery (T4): +110

**Table 3:** Estimated marginal mean (standard error) values and generalized estimating equation (GEE) coefficients for main effects of expectancy condition and expectancy condition by belief interactions involving subjective stress, anxiety, and mood

Main effect: expectancy condition					
		Outcome	df	Wald Chi-square	*p*
Stress		Overall stress post-administration	1	.15	.698
Expect CBD	2.29 (0.14)				
Expect CBD-free	2.57 (0.14)				
Anxiety		Overall anxiety post-administration	1	3.27	.070
Expect CBD	38.70 (0.96)				
Expect CBD-free	41.23 (1.01)				
Negative affect		Overall negative affect post-administration	1	.50	.481
Expect CBD	6.89 (0.20)				
Expect CBD-free	7.04 (0.20)				
Positive affect		**Overall positive affect post-administration**	**1**	**3.92**	**.048**†
Expect CBD	11.29 (0.45)				
Expect CBD-free	12.00 (0.48)				
Interaction: expectancy condition by belief					
		Overall stress post-administration	1	.69	.406
		**Overall anxiety post-administration**	**1**	**5.81**	**.016**
		Overall negative affect post-administration	1	.98	.321
		Overall positive affect post-administration	1	2.45	.118

Bolded coefficients indicate statistical significance (*p* < .05)

†False discovery rate threshold > 5% exceeded (adjusted *p* = .096) indicates a potential false positive finding

*Note.* Overall scores post-administration includes all three timepoints following oil self-administration (i.e., post-absorption, post-stress, recovery)

We first evaluated whether there were differences in subjective intoxication, relaxation, sedation, and stimulation according to expectancy condition (Table 2). No significant Time by Expectancy condition interactions were observed for intoxication, relaxation, and stimulation; however, there were differences in subjective sedation. In the CBD expectancy condition, sedation *increased* significantly from baseline to post-absorption (*p* = .007). In fact, post-absorption sedation was higher in the CBD expectancy condition relative to the CBD-free expectancy condition (*p* = .002). Alternatively, in the CBD-free expectancy condition, subjects reported *lower* levels of sedation during recovery relative to post-stress (*p* = .019) and baseline (*p* = .037).

**Fig. 1:**
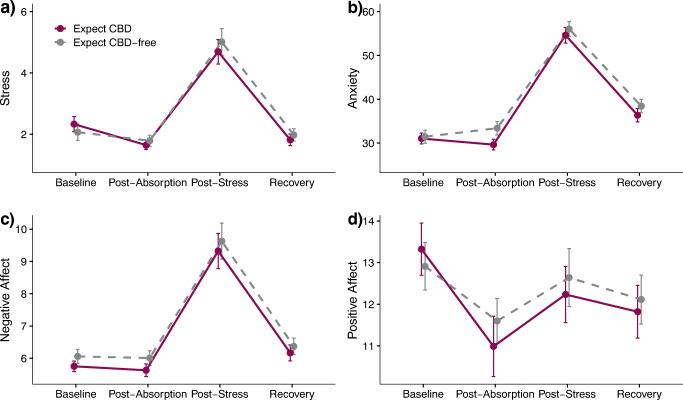
Estimated marginal mean (± standard error). **a** Stress [Numeric Rating Scale “Stress”; score range 1–10], **b** Anxiety [State-Trait Anxiety Inventory- State Version, Short Form; total score range: 20–80]. **c** Negative affect [International Positive Negative Affect Schedule- Short Form; total score range: 5–25]. **d** Positive affect [International Positive Negative Affect Schedule- Short Form; total score range: 5–25] ratings over Time by Expectancy condition. No significant Time by Expectancy condition interactions were observed for any of the subjective ratings. Baseline (T1): +00; Post-absorption (T2): +95; Post-stress (T3): +110; Recovery (T4): +120

Next, we examined whether CBD expectancy alone would dampen subjective stress, anxiety, and mood responses to an acute laboratory stressor (Table 2). First, main effects of time indicated that the MAST was effective at inducing subjective stress, anxiety, and negative affect among all subjects, regardless of expectancy condition (baseline vs. post-stress, all *p* < .001). See Supplemental files for a breakdown of findings related to main effects of time. None of the Time by Expectancy condition interactions reached statistical significance (Fig. 1).

**Fig. 2:**
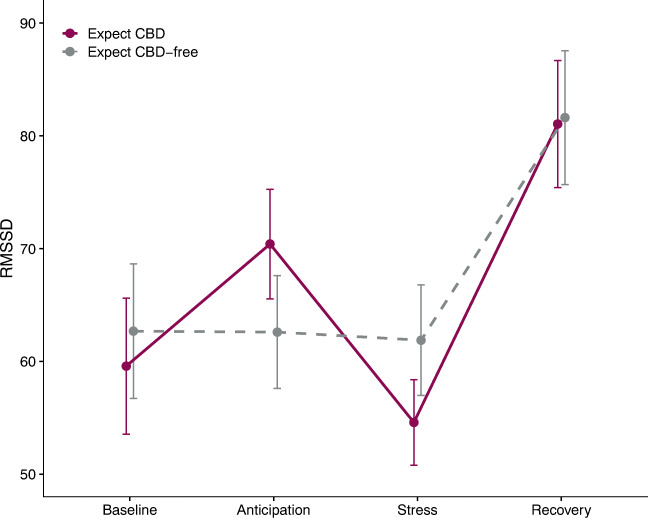
Estimated marginal mean (± standard error) RMSSD, an index of heart rate variability, over Time by Expectancy condition. RMSSD is a measure of vagal tone, thus higher RMSSD is thought to represent more parasympathetic output. A decrease in RMSSD is thought to represent a greater physiological stress response. In the CBD expectancy condition, RMSSD changed sequentially over time, and was higher at recovery relative to baseline. In the CBD-free expectancy condition, RMSSD was lower during stress relative to recovery, and higher at recovery relative to baseline. RMSSD root mean square of successive differences. Baseline (T1): +00 − + 70; Anticipation (T2): +95; Stress (T3): +100; Recovery (T4): +110

To test whether expectancy influenced physiological markers of acute stress, we evaluated Time by Expectancy condition interactions predicting HR and RMSSD, a time-domain index of HRV (Table 2). First, main effects of time were observed for both HR and RMSSD, which tended to change significantly from anticipation to stress (HR increase, *p* = .020; RMSSD decrease, *p* = .002), indicating that the MAST was successful at inducing physiological stress. See supplemental files for a breakdown of findings related to main effects of time. No Time by Expectancy condition interaction was observed for HR; however, a significant interaction was observed for RMSSD (Fig. 2). Parasympathetic nervous system activity dominates RMSSD (Laborde et al. 2017); thus, lower mean RMSSD is thought to represent a larger stress response. In the CBD expectancy condition, RMSSD increased significantly from baseline to anticipation (*p* = .007), then decreased during stress (*p* < .001), and subsequently increased at recovery (*p* < .001). RMSSD was significantly higher at recovery, relative to baseline (*p* < .001). On the other hand, in the CBD-free expectancy condition, RMSSD was comparable during baseline, anticipation, and stress. However, similar to the CBD expectancy condition, RMSSD was lower during stress relative to recovery in the CBD-free expectancy condition (*p* < .001). RMSSD during recovery was also higher than baseline (*p* < .001).

Next, to explore the possibility that expectancy-related influences on subjective stress, anxiety, and mood responses were supressed by the stress task, we evaluated whether expectancy condition influenced *overall* stress, anxiety, and mood ratings following oil administration (i.e., post-absorption, post-stress, and recovery) (Table 3). A main effect of expectancy condition was observed for positive affect such that higher overall ratings of positive affect were reported when participants expected CBD-free versus CBD-containing oil. However, the FDR for this finding exceeded the 5% threshold, suggesting that it may have been a false positive. No significant main effects of expectancy condition were identified for any other subjective rating, indicating that they did not differ by expectancy condition.

**Fig. 3:**
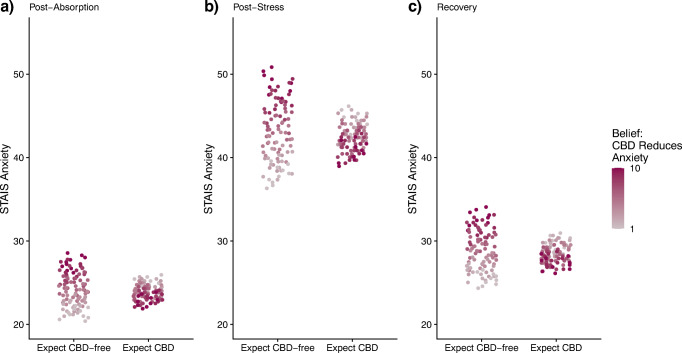
Plot of generalized estimating equation (GEE) model predicted values for ratings of anxiety [State-Trait Anxiety Inventory-State Version, Short Form; total score range: 20–80] (adjusted for baseline scores) by expectancy condition at **a** post-absorption, **b** post-stress, and **c** recovery. Darker points indicate higher endorsement of belief that CBD reduces anxiety, whereas lighter points indicate lower endorsement of belief [Numeric Rating Scale “CBD reduces anxiety”; score range 1–10]. A belief by expectancy condition interaction was observed such that participants who endorsed the highest beliefs that CBD reduces anxiety (third tercile; 9–10) had significantly lower anxiety ratings in the CBD expectancy condition relative to CBD-free condition (across all three time points post-administration). Baseline (T1): +00; Post-absorption (T2): +95; Post-stress (T3): +110; Recovery (T4): +120

To assess whether *a priori* beliefs about CBD effects on stress, anxiety, and mood differentially impacted subsequent stress, anxiety, and mood responses, we explored interactions between belief ratings and expectancy condition. Beliefs about the potential effects of CBD are illustrated in the Online Resource (Supplemental Figure 1). Briefly, stress, anxiety, and mood belief ratings were negatively skewed such that the majority of participants endorsed beliefs closer to the upper end of the scale (i.e., 10). A significant belief by expectancy condition interaction was observed for subjective ratings of anxiety (Fig. 3). Post-hoc tests of simple effects in R indicated that endorsing stronger beliefs that CBD reduces anxiety (third tercile [range: 9–10]) impacted overall anxiety levels according to expectancy condition (*p* = .009). Specifically, those who endorsed higher *a priori* beliefs that CBD reduces anxiety reported significantly less anxiety when they were led to expect CBD oil than when they were led to expect CBD-free oil. Those who endorsed lower belief ratings (first tercile [range: 1–6] and second tercile [range: 7–8]) reported similar anxiety levels across expectancy condition. No significant belief by expectancy condition interactions were observed for overall ratings of stress or mood.

## Discussion

This was the first study to our knowledge to experimentally manipulate and evaluate the effects of CBD-related expectancy. Prior research has shown that CBD administration dampens anxiety and stress responses in humans (Zuardi et al. 1993, 2017; Bergamaschi et al. 2011; Masataka 2019); however, it is unclear whether such effects result from pharmacological properties and/or CBD-related expectancy (i.e., the placebo effect). We were therefore specifically interested in examining whether CBD expectancy, independent from pharmacology, could impact acute stress, anxiety, and mood in a sample of healthy adults. Overall, findings suggested that expectation likely plays some role in the purported stress- and anxiety-reducing effects of CBD.

Various subjective and physiological indices of stress, anxiety, and mood were measured across four time points during each of the two laboratory sessions (expect CBD vs. expect CBD-free). With regard to HRV, we found that the pattern of RMSSD values differed significantly according to expectancy condition. In the CBD-free expectancy condition, RMSSD only changed significantly (i.e., increased) from stress to recovery. Conversely, in the CBD expectancy condition, RMSSD increased significantly during the anticipation to stress, then decreased significantly during stress and increased again at recovery. There are a number of interpretations that could explain this finding. First, anticipation-induced, but not stress-induced changes in HRV have been associated with hypothalamic-pituitary adrenal axis reactivity (Pulopulos et al. 2018), wherein better anticipatory stress regulation (reflected in less HRV decrease during stress anticipation) is thought to represent enhanced overall physiological stress response regulation. Our finding could therefore suggest that CBD expectancy, independent from pharmacology, may dampen physiological indices of stress. Second, the observed fluctuations in HRV in the CBD expectancy condition could indicate a pattern of adaptive emotional responding or normal physiological processes (Porges 1995; Thayer et al. 2012). For instance, challenges that disrupt homeostasis require the individual to respond appropriately (i.e., via the autonomic nervous system) to maintain homeostasis (for a review, see Kim et al. 2018). This could be reflected through fluctuations in vagal output from anticipation to stress, and then from stress to recovery. Third, cannabis use has been shown to increase HRV (Schmid et al. 2010). It is therefore possible that expecting CBD was sufficient to induce similar effects to the drug itself such that HRV increased during anticipation (i.e., post-product absorption). Alternatively, the observed pattern of physiological findings could indicate that CBD expectancy alone dampens physiological indices of stress during anticipation, as illustrated by the significant increase in HRV from baseline to stress anticipation. However, during the stressor itself, there appears to be the opposite effect such that HRV decreases significantly from anticipation to stress. Interestingly, neither of these patterns were observed in the CBD-free expectancy condition. It is therefore possible that participants showed a typical placebo response initially (i.e., lower physiological stress) in the CBD expectancy condition; however, when confronted with the actual stressor, a significant stress response was still elicited.

Interestingly, the lack of change in HRV from baseline to anticipation to stress in the CBD-free expectancy condition suggests that a significant physiological stress response may not have been elicited. It is possible that our baseline assessment of HRV in both conditions may have been confounded by the nature of our study design. Specifically, participants were told at the beginning of each session that they would be randomly assigned to an oil and a task that would either be physically and cognitively demanding, or non-demanding, but they would not know which condition they were assigned to until immediately before. This may have elicited some degree of anticipatory stress and/or anxiety that impacted their baseline/resting HRV assessment.

Among all subjects, the stress task induced self-reported stress, anxiety, and negative affect. Both expectancy conditions appeared to be similar in their subjective stress responses, which seems to partially contradict the physiological data and would lend support to the notion that CBD expectancy does not impact stress and anxiety. However, our findings that the stressor reliably increased subjective indices of stress and anxiety could also suggest a potential ceiling effect wherein the strength of the stressor (comprised of physical, mental, and social challenges) suppressed any expectancy-driven influences. It is also possible that rapid expectancy-induced changes in affective state are more difficult to capture, especially when such changes are small (Campbell and Ehlert 2012). Alternatively, there may be other individual difference factors that interact with expectancy condition to predict subjective stress and anxiety responses.

To explore the possibility that expectancy-related influences on subjective stress, anxiety, and mood responses were being supressed by the stress task, we evaluated differences in subjective affect ratings following oil administration (i.e., post-absorption, post-stress, and recovery). Only positive affect differed according to expectancy condition such that those in the CBD expectancy condition reported *less* positive affect compared to the CBD-free expectancy condition. However, this finding may have been a false positive as indicated by the > 5% FDR. Nevertheless, it would not be particularly surprising that the CBD expectancy condition is associated with lower positive affect given that one of the reported side effects of CBD is sedation (Iffland and Grotenhermen 2017), and some of the positive affect items appeared to be related to physiological arousal (e.g., “Alert,” “Active”). A significant interaction involving subjective sedation supports this explanation, as subjects in the expect CBD condition reported higher levels of sedation post-absorption relative to those in the expect CBD-free condition. Combined with data from the manipulation check indicating that all subjects reported perceived oil contents consistent with instructions during 100% of sessions, the difference in subjective sedation between expectancy groups suggests that the instruction manipulation was indeed successful.

Our findings generally supported the idea that affective responses can be elicited or amplified by the mere expectation of their occurrence (Kirsch 2018). While the majority of participants endorsed moderate-to-high beliefs that CBD was effective at reducing stress, anxiety, and improving mood, their level of endorsement varied widely (i.e., from 1 to 10). Interestingly, the extent to which participants believed that CBD reduced anxiety interacted with expectancy condition to predict their subjective anxiety levels following oil administration (post-absorption, post-stress, recovery). That is, subjects who endorsed the strongest beliefs that CBD reduces anxiety tended to experience the lowest levels of anxiety when they expected CBD oil and the highest levels of anxiety when they expected CBD-free oil. On the other hand, when subjects endorsed low or moderate beliefs, there was very little difference in anxiety outcomes according to expectancy condition. Such findings emphasize the importance of individual expectancies and their role in moderating the placebo effect. They are also consistent with prior research demonstrating that expectations regarding the success of treatment (or effects of a substance) are paramount in predicting treatment (or substance administration) outcomes (Schedlowski et al. 2015). Lastly, there was no observed association between *a priori* stress- and mood- related CBD beliefs and respective subjective outcomes. It is possible that these expectancy effects may be specific to anxiety. Alternatively, the assessment used to measure anxiety may have been more sensitive to short-term affective changes, relative to the subjective stress and mood assessments.

Findings should be considered in light of the following methodological considerations. First, our sample was a relatively homogenous population of healthy, mostly white adults with college or university education, thus limiting our study’s generalizability. Moreover, because we used a sample of healthy adult participants, it is not clear the extent to which our findings would extend to individuals suffering from stress- and anxiety-related conditions for which CBD is often considered. We were also likely underpowered to examine sex-related effects. Additionally, though we were interested in making population level inferences, the use of GEE as an analytic strategy with less than 40 clusters can yield biased results (Kauermann and Carroll 2001). This could be a possibility in our study but is unlikely given our cluster size (i.e., 43) exceeded this threshold. Lastly, since CBD-free hempseed oil was administered to all participants, we could only make inferences about the role of CBD expectancy alone, on various stress, anxiety, and mood responses. Future studies would benefit from using a full balanced-placebo research design (Rohsenow and Marlatt 1981), such that more inferences could be made about whether CBD pharmacology interacts with expectancy or whether CBD pharmacology alone has stress and/or anxiety-dampening effects.

Overall, the present findings provided mixed support towards the first hypothesis that the CBD expectancy condition would be associated with distinct patterns of subjective and physiological responses relative to the CBD-free expectancy condition. While there were no differences in subjective stress, anxiety, and mood between expectancy conditions, higher levels of sedation were reported in the CBD expectancy condition following absorption relative to the CBD-free expectancy condition. Additionally, compared to the similar HRV response over time in the CBD-free expectancy condition, CBD expectancy was associated with a fluctuating pattern of HRV, possibly indicative of a more adaptive physiological stress response or successful emotional adaptation during stress anticipation (but not during the stress challenge). Consistent with our second hypothesis, only those who had the strongest *a* priori beliefs regarding the anxiety-dampening effects of CBD exhibited decreased subjective anxiety following administration of oil in the CBD relative to CBD-free expectancy conditions. Those with lower *a priori* beliefs about the anxiolytic properties of CBD did not show any effects of expectancy condition on their ratings of anxiety. Contrary to our hypothesis, however, no significant effects were identified for mood- or stress-related belief models. Our findings demonstrate, for the first time, that expectancy-related factors likely play a key role in the purported anxiolytic effects of CBD, at least among those who *believe* that it is helpful for such purposes. Our results also provide novel insight into the mechanisms through which CBD may be facilitating medicinal effects for stress- and anxiety-related psychiatric conditions (e.g., Blessing et al. 2015). Future research would also benefit from evaluating the influence of CBD-related expectancy effects in clinical populations and replicating these findings in a larger sample such that sex differences could also be evaluated. Though previous reports suggest that CBD may be a promising medicine for psychiatric disorders like anxiety, our findings emphasize the need for more research evaluating the relative contributions of pharmacological and non-pharmacological factors for such conditions, which could be done through a full balanced placebo research design (Rohsenow and Marlatt 1981). These findings also highlight the need to evaluate and control for *a priori* CBD expectancies in gold standard randomized controlled clinical trials. Lastly, given the dramatic increase in the use of CBD for psychiatric conditions (World Health Organization (WHO) 2018) (despite the dearth of strong empirical support), and the beliefs about its efficacy as demonstrated through our findings, it may be beneficial to allocate resources towards education-focused initiatives to correct these misperceptions that are accessible to the lay public.

## Supplementary information

ESM 1 (EPS 64 kb)

ESM 2 (DOCX 28 kb)
