# Supplementary material for: Evaluating cannabidiol (CBD) expectancy effects on acute stress and anxiety in healthy adults: a randomized crossover study
Source: Psychopharmacology (Berl). 2021 Apr 4;238(7):1965–77. doi: 10.1007/s00213-021-05823-w (PMC8233292; doi:10.1007/s00213-021-05823-w)
Supplement: Supplementary file 2 — (DOCX 28 kb) [file 213_2021_5823_MOESM2_ESM.docx]

**Online Supplemental Files**

**Main effects of Time**

See Supplemental Table 1 for generalized estimating equation (GEE) coefficients as well as estimated marginal means and standard error values for all main effects of Time. First, main effects of Time were observed for ratings of sedation, stimulation, intoxication, and relaxation. Subjective intoxication and sedation ratings increased from baseline to post-absorption (*p*=.008, *p*=.043, respectively), while stimulation ratings decreased from baseline to post-absorption (*p*<.001). Sedation then decreased from post-stress to recovery (*p*=.041). At baseline (relative to post-stress and recovery), intoxication was rated lowest (both *p*=.01) while stimulation was rated highest (both *p*<.001). Additionally, subjective relaxation was lowest post-stress relative to all other time points (baseline, post-absorption, and recovery; all *p*<.001). Subjects also reported higher ratings of relaxation at baseline relative to recovery (*p*<.001).

Next, main effects of Time indicated that the MAST was effective at inducing subjective stress, anxiety, and negative affect (baseline vs. post-stress, all *p*<.001) among all subjects, regardless of expectancy condition. Only ratings of stress decreased from baseline to post-absorption (*p*<.001). All subjective ratings decreased significantly from post-stress to recovery (all *p*<.001). Ratings of anxiety and negative affect were higher at recovery relative to baseline (*p*<.001, *p*=.029, respectively). A main effect of Time was also observed for positive affect. Ratings of positive affect decreased from baseline to post-absorption (*p*<.001), then increased from post-absorption to post-stress (*p*=.028). Positive affect was higher at baseline than recovery (*p*=.007).

Lastly, main effects of Time were observed for both time-domain indices of HRV, HR and RMSSD, indicating that the MAST was effective at inducing physiological markers of acute stress. HR increased from baseline to anticipation and stress (both *p*≤.001). RMSSD and HR changed significantly from anticipation to stress (RMSSD decrease, *p*=.002; HR increase, *p*=.020), then from stress to recovery (RMSSD increase, *p*<.001; HR decrease, *p*<.001), indicative of a physiological stress response. Physiological stress during recovery was significantly lower than baseline (RMSSD higher, *p*<.001; HR lower, *p*<.001).

**Tables**

| ***Main effect: Time*** | | | | | | | | | |
| --- | --- | --- | --- | --- | --- | --- | --- | --- | --- |
|  | **Baseline** | **Post-absorption** | **Post-stress** | **Recovery** |  | **Outcome** | **df** | **Wald Chi-square** | ***p*** |
| Intoxication | 1.01(0.01) | 1.19(0.07) | 1.26(0.10) | 1.19(0.08) |  | **Intoxication** | **3** | **10.04** | **.018** |
| Relaxation | 6.26(0.25) | 6.52(0.26) | 2.29(0.23) | 4.66(0.28) |  | **Relaxation** | **3** | **124.36** | **<.001** |
| Stimulation | 14.30(0.63) | 11.64(0.78) | 11.36(0.80) | 12.04(0.77) |  | **Stimulation** | **3** | **44.90** | **<.001** |
| Sedation | 7.55(0.52) | 8.56(0.65) | 8.18(0.64) | 6.96(0.54) |  | **Sedation** | **3** | **17.10** | **.001** |
| Stress | 2.20 (0.22) | 1.72 (0.14) | 4.85 (0.36) | 1.90 (0.16) |  | **Stress** | **3** | **222.76** | **<.001** |
| Anxiety | 31.25 (1.09) | 31.46 (1.08) | 55.33(1.49) | 37.38(1.21) |  | **Anxiety** | **3** | **299.71** | **<.001** |
| Negative affect | 5.90(0.15) | 5.81(0.15) | 9.48 (0.47) | 6.27 (0.21) |  | **Negative Affect** | **3** | **148.49** | **<.001** |
| Positive affect | 13.12(0.54) | 11.29(0.58) | 12.44(0.61) | 11.97(0.58) |  | **Positive Affect** | **3** | **27.41** | **<.001** |
|  | **Baseline** | **Anticipation** | **Stress** | **Recovery** |  |  |  |  |  |
| HR | 68.66(1.10) | 72.08(1.23) | 75.00(1.53) | 63.53(1.10) |  | **HR** | **3** | **148.23** | **<.001** |
| RMSSD | 61.12(5.69) | 66.39(4.51) | 58.12(3.89) | 81.33(5.35) |  | **RMSSD** | **3** | **66.94** | **<.001** |

**Supplemental Table 1.** Estimated marginal mean (standard error) values and generalized estimating equation (GEE) coefficients for main effects of Time involving subjective drug effects, stress, anxiety, and mood, and heart rate variability.

Bolded coefficients indicate statistical significance (*p*<.05).

Subjective measures: Baseline (T1): +00; Post-absorption (T2): +95; Post-stress (T3): +110; Recovery (T4): +120

Physiological measures: Baseline (T1): +00-+70; Anticipation (T2): +95; Stress (T3): +100; Recovery (T4): +110

**Supplemental Table 2.** Generalized estimating equation (GEE) coefficients from factors and covariates included in each model

| ***Covariate: Baseline scores*** | | | |
| --- | --- | --- | --- |
| **Outcome** | **df** | **Wald Chi-square** | ***p*** |
| **Overall stress post-administration** | **1** | **28.89** | **<.001** |
| **Overall anxiety post-administration** | **1** | **24.83** | **<.001** |
| **Overall negative affect post-administration** | **1** | **23.70** | **<.001** |
| Overall positive affect post-administration | 1 | 1.64 | .200 |
| ***Covariate: Belief rating*** |  |  |  |
| **Outcome** | **df** | **Wald Chi-square** | ***p*** |
| Overall stress post-administration | 1 | 2.37 | .124 |
| Overall anxiety post-administration | 1 | .78 | .378 |
| **Overall negative affect post-administration** | **1** | **3.86** | **.049** |
| **Overall positive affect post-administration** | **1** | **19.40** | **<.001** |
| ***Factor: Time*** | | | |
| **Outcome** | **df** | **Wald Chi-square** | ***p*** |
| **Overall stress post-administration** | **2** | **187.86** | **<.001** |
| **Overall anxiety post-administration** | **2** | **332.72** | **<.001** |
| **Overall negative affect post-administration** | **2** | **146.45** | **<.001** |
| **Overall positive affect post-administration** | **2** | **6.75** | **.034** |
| ***Covariate: ECG-derived breathing rate*** | | | |
| **Outcome** | **df** | **Wald Chi-square** | ***p*** |
| **HR** | **1** | **6.86** | **.009** |
| **RMSSD** | **1** | **9.34** | **.002** |
| ***Factor: Expectancy condition*** |  |  |  |
| **Outcome** | **df** | **Wald Chi-square** | ***p*** |
| HR | 1 | .35 | .557 |
| RMSSD | 1 | .15 | .697 |
| Stress | 1 | .14 | .706 |
| **Anxiety** | **1** | **3.94** | **.047** |
| Negative affect | 1 | 2.26 | .133 |
| Positive affect | 1 | .52 | .472 |
| Intoxication | 1 | 2.42 | .120 |
| Relaxation | 1 | .16 | .692 |
| Stimulation | 1 | .12 | .729 |
| Sedation | 1 | 2.41 | .121 |

Bolded coefficients indicate statistical significance (*p*<.05).

*Note.* The coefficients listed in this table were not included in the Benjamini-Hotchberg adjustment as they were not effects of interest.

**Figure Captions**

**Supplemental Fig. 1** Density plots illustrating *a priori* CBD belief ratings on a scale from 1 (not at all) to 10 (completely) for the following items: a) Reduces Stress (M=6.74, SD=1.63), b) Reduces Anxiety (M=6.86, SD=1.95), and c) Improves Mood (M=6.21, SD=1.88). Vertical dashed lines represent mean ratings
